# Outcomes of ME/CFS following infectious mononucleosis: seven-year follow-up of a prospective study

**DOI:** 10.3389/fmed.2026.1676628

**Published:** 2026-02-27

**Authors:** Leonard A. Jason, Jacob Furst, Rebecca Worth, Ben Z. Katz

**Affiliations:** 1 Center for Community Research, DePaul University, Chicago, IL, United States; 2 College of Computing and Digital Media, DePaul University, Chicago, IL, United States; 3 Feinberg School of Medicine, Ann and Robert H. Lurie Children’s Hospital of Chicago, Northwestern University, Chicago, IL, United States

**Keywords:** chronic fatigue syndrome, Epstein Barr virus, infectious mononucleosis, myalgic encephalomyelitis, myalgic encephalomyelitis/chronic fatigue syndrome

## Abstract

**Background:**

Many individuals with Myalgic Encephalomyelitis/Chronic Fatigue Syndrome (ME/CFS) report experiencing an infectious illness prior to disease onset. Approximately 30% of cases are linked to Epstein–Barr virus (EBV) infection resulting in Infectious Mononucleosis (IM).

**Methods:**

We examined the progression of ME/CFS following IM among a cohort of college students who were recruited before they developed the infection. This sample represented a socioeconomically and ethnically diverse population of young adults who were monitored over a 7-year period. Assessments of health status, psychological functioning, and blood biomarkers were conducted at four time points: (1) baseline, when participants were healthy and at least 6 weeks from IM onset; (2) within 6 weeks of IM diagnosis; (3) 6 months post-IM, when participants had either recovered or met criteria for ME/CFS; and (4) the 7-year follow-up.

**Results:**

At follow-up, 81% of participants who had initially presented with severe ME/CFS continued to fulfill diagnostic criteria. In contrast, only about one-third of those with moderate or lingering symptoms at 6 months still had ME/CFS 7 years later.

**Conclusion:**

These findings indicate that ME/CFS following IM tends to persist over the long term, particularly among those whose illness was more severe at onset.

## 1 Introduction

Infectious Mononucleosis (IM) has been implicated in roughly 30% of Myalgic Encephalomyelitis/Chronic Fatigue Syndrome (ME/CFS) cases (1). While most individuals recover from IM within approximately 6 weeks (median duration of illness: 16 days), a subset go on to develop ME/CFS and continue to experience functional limitations (2). Reported rates of ME/CFS following IM vary across studies: White and colleagues (3) observed a 9% incidence at 6 months post-infection; Hickie et al. (4) documented an 11% rate at the same time point; and Katz and colleagues (5) reported rates of 13% and 4% at 6 and 24 months after IM, respectively. These studies represent findings from individuals from different ages, and age can skew the data from ME/CFS research (6).

Few investigations have tracked representative cohorts of individuals who developed ME/CFS across extended periods. For instance, Ciccone, Chandler, and Natelson (7) conducted a 2.5-year follow-up of patients recruited from a tertiary care setting and reported that symptoms generally persisted among those with ME/CFS. Similarly, Jason and colleagues (8) examined socioenvironmental influences and symptom patterns over a decade, finding that levels of disability, fatigue, social support, optimism, and coping remained relatively stable throughout the study period.

Of those studies cited above dealing with IM, they evaluated participants only after Epstein–Barr virus (EBV) infection. In contrast, prospective designs that track individuals prior to infection can offer valuable information about risk factors contributing to chronic illness. Between 2014 and 2018, we enrolled 4,501 college students from diverse demographic backgrounds and obtained baseline data at least 6 weeks before any developed Infectious Mononucleosis (IM; Stage 1). Students were then monitored for IM onset (Stage 2), which occurred in 238 participants (5.3%). At 6 months post-infection (Stage 3), diagnostic evaluations identified 55 of these 238 as meeting criteria for ME/CFS, while 157 were symptom-free (9). Of the recovered group, 67 served as comparison controls. For analysis, participants with severe ME/CFS (defined as meeting more than one diagnostic case definition) were contrasted with those meeting only a single set of criteria (moderate ME/CFS) as well as with recovered controls. The present report extends these findings by presenting seven-year follow-up outcomes from this cohort. We anticipated that participants classified as severely ill would be most likely to exhibit persistent symptoms across time.

## 2 Methods

At Stage 1, students from Northwestern University (NU) were enrolled at least 6 weeks prior to the onset of Infectious Mononucleosis (IM). Diagnosis of IM was based either on a positive monospot test or on evidence of a primary Epstein–Barr virus (EBV) serological response, indicated by the presence of viral capsid antigen (VCA) IgM antibodies and/or VCA IgG accompanied by a negative EB nuclear antigen result, within the clinical presentation of mononucleosis. The Northwestern University Student Health Service (NUHS), along with additional healthcare providers, was utilized to confirm diagnoses and monitor the occurrence of IM in participants.

Following online informed consent, participants completed a battery of questionnaires administered through the Research Electronic Data Capture (REDCap) platform (10), as detailed in the measures section. Students who subsequently developed IM were enrolled within 6 weeks of their diagnosis (Stage 2), at which point they re-consented, completed the same questionnaire set, and provided an additional blood sample. Approximately 5 months post-diagnosis, participants were contacted by phone to assess recovery status. Those reporting unresolved symptoms, along with a comparison group of recovered students matched on age, sex, and academic year at the time of IM, were invited to take part in Stage 3 assessments 6 months after IM onset and Stage 4 assessments 7 years later. At each follow-up, informed consent was obtained again, and participants completed another round of questionnaires. Blood specimens were collected at every stage: serum and plasma at baseline (Stage 1), and serum, plasma, and viable white blood cells at Stages 2 through 4. During Stages 3 and 4, participants also underwent full medical examinations. Additional clinical information was obtained to exclude alternative medical explanations for ME/CFS. Laboratory evaluations followed the criteria outlined by Fukuda et al. (11), with modifications by Reeves et al. (12), to identify exclusionary conditions [see Katz et al. (5)]. The study received ethical approval from the Institutional Review Boards of Northwestern University, DePaul University, and the Stanley Mann Research Institute at Ann & Robert H. Lurie Children’s Hospital of Chicago.

### 2.1 Questionnaires

Compass 31. The Compass 31, derived from the Autonomic Symptom Profile (13), is a validated measure designed to evaluate autonomic dysfunction. It has demonstrated strong reliability and is widely used in research assessing autonomic symptom burden (14).

Medical Outcomes Study 36-Item Short-Form Health Survey (SF-36/RAND). The SF-36 evaluates the extent to which health impacts both physical and psychological functioning, in addition to providing a general index of health-related quality of life (15). Subscale scores are structured so that higher values reflect better functioning. Evidence supports the measure’s internal consistency and discriminant validity (16). We used the Physical Component Health Domain, the General Health and Mental Health subscales.

DePaul Symptom Questionnaire (DSQ). The DSQ is a structured self-report tool for assessing ME/CFS symptoms and illness history (17). It allows for standardized application of multiple case definitions, including Fukuda et al. (11), the Canadian Clinical Criteria (18), the ME-International Consensus Criteria (19), and the Institute of Medicine (IOM) definition (20). Symptoms are rated for both frequency and severity over the preceding 6 months using 5-point Likert scales. Scores are transformed to 100-point values by averaging across frequency and severity, dividing by 2, and multiplying by 25 to create a composite for each symptom. The DSQ has been shown to have good test–retest reliability across patient and control groups (21). Independent validation by Murdock et al. (22) confirmed excellent internal reliability and strong discrimination between cases and controls (23). In addition to individual symptoms, we derived both a total DSQ score and a Fatigue Score by averaging frequency and severity composites.

Fatigue Severity Scale (FSS). The FSS, developed by Krupp et al. (24), consists of nine items scored on a 7-point scale. It emphasizes the behavioral impact of fatigue and is sensitive to variations in symptom intensity. Prior work has demonstrated the FSS’s ability to distinguish individuals with ME/CFS from those with multiple sclerosis (MS) or major depression (24, 25). Normative data are available for populations with MS, systemic lupus erythematosus (SLE), and healthy controls.

Coping Orientation to Problems Experienced (COPE). The COPE inventory measures coping strategies through 28 items rated on a 4-point scale (26). Fourteen distinct coping styles are identified, which can be grouped into three higher-order categories: (a) emotion-focused coping (e.g., emotional support, acceptance, positive reframing, religion, humor); (b) problem-focused coping (e.g., active coping, planning, instrumental support); and (c) dysfunctional coping (e.g., denial, behavioral disengagement, venting, substance use, self-blame, self-distraction) (27). The instrument has been validated and demonstrates acceptable reliability.

Perceived Stress Scale (PSS). The PSS short form includes four items measuring perceived stress over the past month (28). The abbreviated version has a reported internal consistency reliability of 0.72.

Beck Depression Inventory-II (BDI-II). Depressive symptoms were assessed with the BDI-II (29), which has been validated for use in both depressed and non-depressed ME/CFS populations (30).

Beck Anxiety Inventory (BAI). Anxiety was measured using the BAI (31), a 21-item self-report scale characterized by high internal consistency and established reliability.

### 2.2 ME/CFS case definitions

The study applied several established case definitions for ME/CFS. The Fukuda et al. criteria (11) were one of the primary diagnostic standards. Reliable methods (32) were used to determine substantial reduction in functioning for all case definitions used in this study. In addition, the Canadian Clinical Criteria (18) and the Institute of Medicine (IOM) definition (20) were also employed. Symptom severity was categorized based on the number of case definitions met: participants meeting only one set of criteria were classified as having Moderate ME/CFS, whereas those meeting more than one case definition were categorized as Severe ME/CFS. In general, those meeting the moderate category only met the Fukuda criteria. Students who underwent Stage 3 evaluation but did not fulfill ME/CFS criteria and nevertheless continued to experience symptoms were classified as having Persisting symptoms. These individuals still had symptoms but might not have had enough symptoms or limitations in functioning to meet one of the case definitions.

### 2.3 Statistical analysis

Survey data were analyzed using a mixed-model analysis of variance to examine differences across the four participant groups (Severe ME/CFS, Moderate ME/CFS, Persisting Symptoms, and Recovered Controls) at each of the four study stages. When appropriate, post-hoc pairwise comparisons were conducted using either Bonferroni correction or the Games-Howell procedure for cases with unequal variances. To account for multiple comparisons, statistical significance was set at *p* < 0.01. For each instrument, we evaluated the main effects of time (Stages 1–4) and group, as well as the interaction between Time and Group, on the outcome measures.

### 2.4 Result**s**

**Figure 1:**
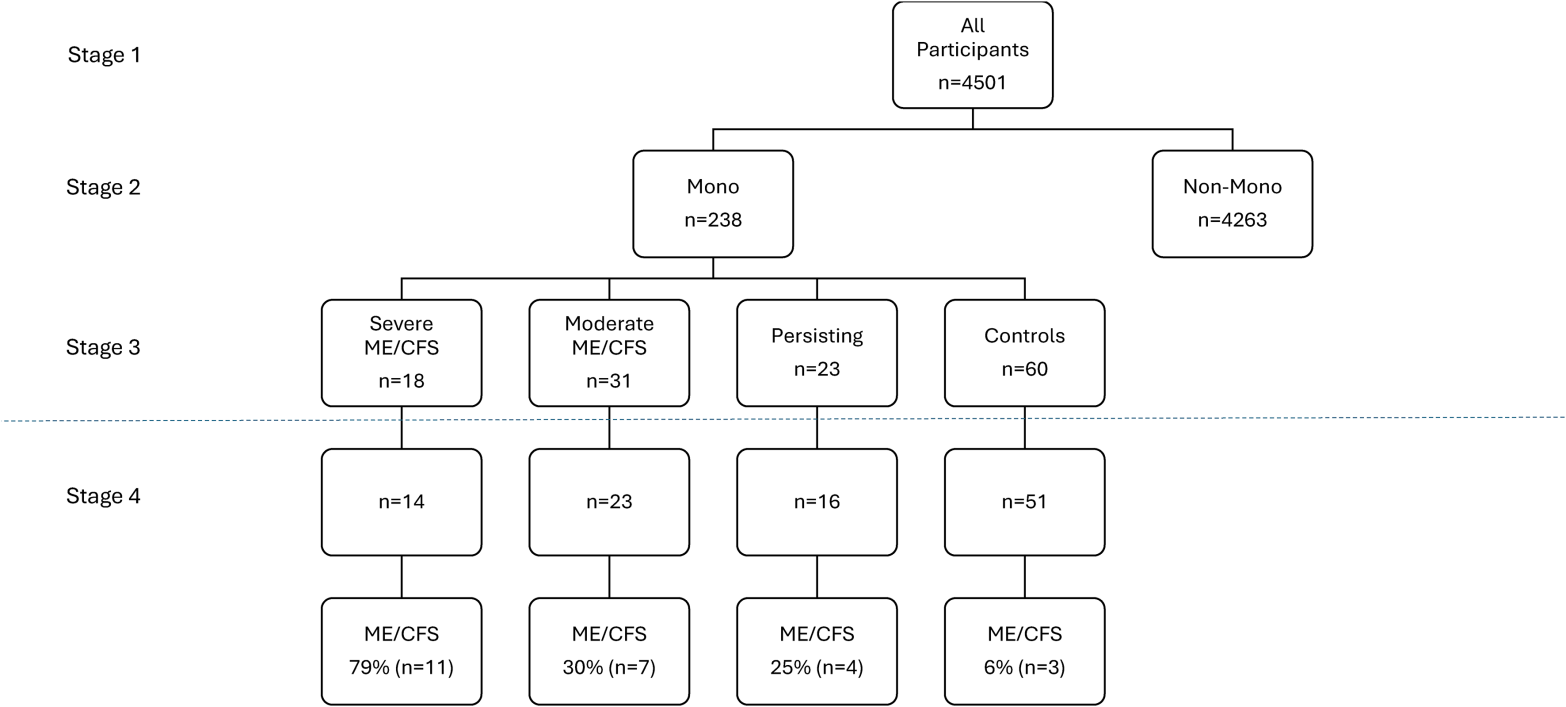
Longitudinal data over time.

Data just includes those where we have Stage 1 baseline data. Figure 1 shows that of the 18 students with severe ME/CFS at Stage 3, we were able to collect data from 78% of them (*n* = 14) at the 7-year follow-up. For the 31 students with moderate ME/CFS at Stage 3, we were able to collect data from 74% of them (*n* = 23) at Stage 4. There were also 23 individuals at Stage 3 with Persisting symptoms, and 70% (*n* = 16) of them were available for Stage 4. Finally, among the 60 recovered controls at Stage 3, 85% (*n* = 51) were available at Stage 4. There were no significant overall differences in the recruitment of Stage 4 participants vs. those who could not be recruited at Stage 4 in terms of age, gender, and racial/ethnic mix. Within these four groups, at Stage 4, we found an ME/CFS diagnosis for 79% (*n* = 11) who had Severe ME/CFS at Stage 3; 30% (*n* = 7) in those with Moderate ME/CFS at Stage 3; 25% (*n* = 4) of those with Persisting Symptoms at Stage 3, and 6% (*n* = 3) who were Recovered at Stage 3. There was a significant difference in these rates of ME/CFS across the four groups, x^2^(*N* = 104) = 9.82, *p* < 0.05.

Statistically significant sociodemographic differences were not found among the three diagnostic groups (see Table 1). The percentage of females and ethnic/racial mix of subjects in the study roughly correlated with the percentages present in each class at Northwestern.

**Table 1:** Demographics for mono groups.

	Severe ME/CFS *N* = 14	Moderate ME/CFS *N* = 23	Persisting *N* = 16	Control *N* = 51
	M (SD)	M (SD)	M (SD)	M (SD)
	% (*n*)	% (*n*)	% (*n*)	% (*n*)
Age (yrs)				
Initial	18.9 (0.6)	19.0 (1.0)	18.8 (0.6)	19.1 (0.7)
Stage 4	26.2 (1.3)	26.0 (1.5)	26.1 (1.4)	26.2 (13.3)
Gender				
Female	50 (8)	78 (18)	81 (13)	56 (28)
Male	43 (6)	23 (5)	19 (3)	44 (22)
Race				
White	71 (10)	83 (19)	81 (13)	78 (40)
Black	14 (2)	9 (2)	6 (1)	12 (6)
Asian	0 (0)	4 (1)	0 (0)	4 (2)
Other	14 (2)	0 (0)	0 (0)	0 (0)
More 1	0 (0)	4 (1)	13 (2)	6 (3)

COMPASS 31. There was a main effect of group [*F* (3, 96) = 12.47, *p* < 0.01], time [*F* (3, 288) = 14.57, p < 0.01], and interactions [*F* (9, 288) = 3.30, p < 0.01] for Compass 31 outcomes (See Table 2 for self-report measures). At Stage 1, there were no significant differences between the four groups. At Stage 2, the Severe group scored worse than the Control group. At Stage 3, the Severe, Moderate, and Persisting groups scored worse than the Control. There were no significant differences at Stage 4.

**Table 2:** Summary and domain scores of survey measures over four stages.

Measures	Stage 1	Stage 2	Stage 3	Stage 4
	M (SD)	M (SD)	M (SD)	M (SD)
Compass 31				
S-ME/CFS	18.93 (15.25)	34.77 (9.54)^a^	31.11 (14.88)^a^	19.50 (19.20)
M-ME/CFS	14.96 (8.30)	22.06 (13.11)	23.31 (10.28)^b^	17.84 (12.64)
Persisting	16.83 (12.61)	23.72 (13.64)	21.31 (12.42)^c^	14.20 (13.58)
Controls	12.51 (10.73)	14.26 (10.93)^a^	10.04 (9.19)^a,b,c^	11.23 (9.46)
SF-36				
Physical composite				
S-ME/CFS	53.59 (7.40)	38.08 (6.59)	42.16 (9.36)^a,e^	50.81 (8.00)
M-ME/CFS	54.92 (5.34)	40.63 (8.47)	49.09 (6.85)^b^	52.24 (8.78)
Persisting	53.72 (8.75)	40.48 (5.66)	53.54 (7.21)^e^	52.78 (8.48)
Controls	54.73 (5.76)	43.50 (7.65)	56.14 (3.96)^a,b^	57.20 (4.92)
General health				
S-ME/CFS	66.82 (17.93)	52.64 (14.89)^a^	43.73 (18.15)^a,e^	59.09 (19.37)^a^
M-ME/CFS	66.25 (19.54)	60.55 (19.80)	59.30 (18.17)^b^	69.40 (23.84)
Persisting	71.92 (10.56)	69.33 (14.61)	70.17 (13.29)^e^	67.50 (22.95)
Controls	73.78 (13.20)	71.25 (14.06)^a^	76.46 (2.19)^a,b^	79.87 (15.09)^a^
Mental health				
S-ME/CFS	54.55 (16.62)^a^	40.73 (23.10)^a^	48.00 (17.16)^a^	56.73 (20.69)
M-ME/CFS	64.83 (18.70)	55.81 (18.91)	53.90 (16.33)^b^	65.33 (16.31)
Persisting	71.67 (16.04)	61.67 (25.72)	65.67 (15.86)	59.33 (21.50)
Controls	73.04 (12.63)^a^	70.96 (13.02)^a^	75.13 (13.25)^a,b^	69.48 (14.52)
DSQ				
Total				
S-ME/CFS	22.82 (9.56)^a^	37.87 (9.84)^a,d^	39.25 (12.73)^a,d,e^	24.51 (10.28)^a^
M-ME/CFS	16.49 (8.11)	25.90 (9.67)^b,d^	19.26 (8.61)^b,d^	15.68 (8.99)^b^
Persisting	14.42 (5.62)	28.31 (12.52)^c^	17.64 (8.54)^c,e^	16.35 (11.37)^c^
Controls	12.77 (7.23)^a^	17.03 (8.23)^a,b,c^	8.99 (5.00)^a,b,c^	8.61 (5.60)^a,b,c^
Fatigue				
S-ME/CFS	38.39 (21.06)	55.34 (21.21)^a^	71.43 (17.97)^a,d,e^	56.25 (13.65)^a,d^
M-ME/CFS	35.94 (18.91)	50.00 (16.89)^b^	42.71 (15.61)^b,d^	29.17 (22.92)^d^
Persisting	30.15 (13.30)	52.94 (17.97)^c^	41.91 (13.21)^c,e^	36.76 (23.58)^c^
Controls	26.02 (15.27)	35.20 (17.98)^a,b,c^	23.47 (12.66)^a,b,c^	15.81 (14.83)^a,c^
FSS				
S-ME/CFS	34.00 (8.17)	50.36 (9.73)^a^	45.64 (11.78)^a,e^	38.18 (9.91)^a^
M-ME/CFS	33.65 (12.36)	43.45 (13.11)	39.30 (9.14)^b^	31.00 (11.84)
Persisting	28.27 (13.38)	44.46 (9.66)	29.18 (10.86)^e^	29.18 (12.94)
Controls	27.89 (10.79)	36.24 (11.75)^a^	22.87 (9.03)^a,b^	22.89 (10.78)^a^
COPE				
Dysfunctional				
S-ME/CFS	18.64 (5.80)	21.55 (6.39)	19.82 (5.02)	21.55 (7.10)^a^
M-ME/CFS	20.45 (5.30)	22.35 (5.44)	21.25 (5.28)	18.65 (3.42)
Persisting	18.00 (4.43)	19.64 (3.96)	19.27 (4.41)	19.00 (4.22)
Controls	18.44 (3.67)	17.61 (3.15)	17.59 (3.67)	17.00 (3.30)^a^
Emotion focused				
S-ME/CFS	21.46 (7.42)	22.55 (5.20)	20.64 (6.89)	23.27 (5.83)
M-ME/CFS	22.50 (4.77)	22.50 (3.71)	21.00 (5.44)	20.85 (5.54)
Persisting	20.09 (5.54)	22.91 (5.34)	21.73 (5.16)	23.82 (5.17)
Controls	21.00 (5.13)	21.26 (4.74)	20.33 (5.37)	20.59 (6.13)
Problem focused				
S-ME/CFS	12.53 (4.29)	13.29 (4.92)	13.65 (4.66)	13.65 (4.27)
M-ME/CFS	14.00 (3.76)	14.75 (3.85)	15.18 (5.48)	14.64 (4.05)
Persisting	13.95 (4.52)	13.90 (4.48)	14.86 (4.42)	14.71 (5.05)
Controls	14.52 (3.94)	14.30 (3.64)	14.87 (4.53)	13.85 (3.92)
PSS				
S-ME/CFS	7.73 (2.80)	9.18 (2.60)	8.91 (2.91)^a^	6.55 (3.42)
M-ME/CFS	6.65 (3.69)	8.65 (2.52)	7.95 (3.02)^b^	5.70 (3.36)
Persisting	4.91 (2.70)	6.55 (4.61)	5.91 (3.42)	5.82 (4.26)
Controls	5.74 (2.91)	6.07 (2.95)	5.24 (2.52)^a,b^	5.09 (2.32)
BDI-II				
S-ME/CFS	12.09 (7.88)	24.00 (13.81)^a^	20.73 (10.38)^a,e^	15.82 (10.49)^a^
M-ME/CFS	10.10 (9.26)	17.57 (11.49)^b^	15.00 (9.74)^b^	7.62 (6.22)
Persisting	7.91 (8.17)	14.55 (13.28)	9.09 (9.82)^e^	8.91 (10.91)
Controls	5.76 (4.61)	7.96 (6.52)^a,b^	4.07 (4.49)^a,b^	4.11 (4.62)^a^
BAI				
S-ME/CFS	8.55 (6.20)	17.82 (9.14)^a^	14.64 (9.63)^a^	9.64 (4.82)^a^
M-ME/CFS	9.38 (7.70)^b^	15.29 (10.12)^b^	10.38 (8.69)^b^	7.00 (5.79)
Persisting	5.82 (3.76)	13.18 (10.08)	7.73 (4.84)	8.64 (8.97)
Controls	4.35 (4.85)^b^	6.22 (7.05)^a,b^	3.09 (3.47)^a,b^	2.98 (4.05)^a^

Similar supescript letters in columns of outcome variables indicate significant differences. ^a^ = *p* < 0.01, Severe vs. Controls. ^b^ = *p* < 0.01, Moderate vs. Controls. ^c^ = *p* < 0.01, Persisting vs. Controls. ^d^ = *p* < 0.01, Severe vs. Moderate. ^e^ = *p* < 0.01, Severe vs. Persisting. ^f^ = *p* < 0.01, Moderate vs. Persisting.

SF-36. For the Physical Component Health domain, there was a main effect for group [*F* (3, 85) = 9.99, *p* < 0.01], time [*F* (3, 255) = 65.93, p < 0.01], and interactions [*F* (9, 255) = 3.04, *p* < 0.01]. There were no significant Physical Health domain differences at Stage 1 or Stage 2 but at Stage 3, the Severe group had worse scores than the Persisting and Control groups, and the Moderate group had worse scores than Controls. By Stage 4, there were no longer significant differences between the conditions. For the General Health domain, there was a main effect of the group [*F* (3, 85) = 9.55, *p* < 0.01], time [*F* (3, 255) = 6.03, *p* < 0.01], and interactions [*F* (9, 255) = 2.86, *p* < 0.01]. There were no significant differences among the three groups at Stage 1 for this domain. For Stage 2, the Severe group was more impaired than the Control group. For Stage 3, the Severe group was worse than the Control and Persisting groups, the Moderate group was worse than the Control group. By Stage 4, only the Severe group was worse than the Controls. For the Mental Health domain, there was a main effect of the group [*F* (3, 85) = 11.29, *p* < 0.01], time [*F* (3, 255) = 5.47, *p* < 0.01], and interactions [*F* (9, 255) = 3.08, *p* < 0.01]. There was a significant differences between the Severe and the Control groups at Stage 1 for this domain. The Severe ME/CFS group was significantly worse than the Controls at Stage 2. At Stage 3, the Severe group was significantly worse than the Controls, and the Moderate group was significantly worse than Controls. At Stage 4, there were no significant differences among the groups.

DePaul Symptom Questionnaire. For the Total DSQ score, there was a main effect of group [*F* (3, 99) = 38.49, *p* < 0.01], time [*F* (3, 297) = 51.05, *p* < 0.01], and interactions [*F* (9, 297) = 7.30, *p* < 0.01]. For Stage 1, the Severe group scored worse than the Controls. At Stage 2, the Severe, Moderate, and Persisting groups scored worse than the Controls; and the Severe was worse than the Moderate. At Stage 3, the Severe group scored worse than the other three groups and the Moderate and Persisting groups scored worse than the Controls. At Stage 4, the Severe, Moderate, and Persisting groups scored worse than the Controls.

**Figure 2:**
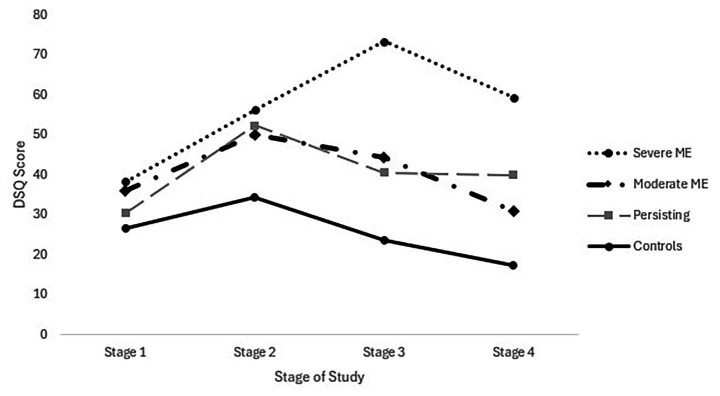
Fatigue across four stages.

For the Fatigue DSQ score, there was a main effect of the group [*F* (3, 100) = 33.00, *p* < 0.01], time [*F* (3, 300) = 21.63, *p* < 0.01], and interactions [*F* (9, 300) = 5.18, *p* < 0.01]. For Stage 1, there were no significant differences across conditions. For Stage 2, the Severe, Moderate, and Persisting groups scored worse than Controls. For Stage 3, the Severe group scored worse than the other three groups, and the Moderate and Persisting groups scored worse than the Controls. At Stage 4, the Severe group scored worse than the Moderate and Controls, and the Persisting group scored worse than the Controls. Figure 2 shows these scores over time.

Fatigue Severity Scale. There was a main effect of group [*F* (3, 84) = 15.43, *p* < 0.01], time [*F* (3, 252) = 27.29, *p* < 0.01], and interactions [*F* (9, 252) = 2.65, *p* < 0.01] for the FSS. At Stage 1, there were no significant differences. At Stage 2, the severe group had worse scores than the Controls. At Stage 3, the severe group had worse scores than the Controls and Persisting groups; the Moderate group also had worse scores than the Controls. At Stage 4, the severe group had worse scores than the Controls.

COPE. There were no main effects for either emotion-focused or problem-focused coping strategies between the four groups. For dysfunctional coping, there was a main effect of group [*F* (3, 84) = 4.90, *p* < 0.01], but not for time or interaction.

Perceived Stress Scale. There was a main effect of group [*F* (3, 84) = 5.10, *p* < 0.01] and time [*F* (3, 252) = 8.73, *p* < 0.01] for the PSS; however, there was no significant interaction. There were no significant differences between the four groups at Stage 1 or 2. At Stage 3, the Severe and Moderate groups had higher scores than the Controls. At Stage 4, there were no significant differences between groups.

Beck’s Depression Inventory II. There was a main effect of group [*F* (3, 85) = 14.72, *p* < 0.01], time [*F* (3, 255) = 24.05, p < 0.01], and interactions [*F* (9, 255) = 4.05, *p* < 0.01] for the BDI-II. At Stage 1, there were no significant differences between the four groups. At Stage 2, the Severe and Moderate groups had higher scores than the Controls. At Stage 3, the severe group had higher scores than the Persisting and Control groups. The Moderate group had higher scores than the Controls. At Stage 4, the severe group had higher scores than the Controls.

Beck’s Anxiety Inventory. There was a main effect of group [*F* (3, 85) = 15.28, *p* < 0.01] and time [*F* (3, 255) = 18.95, *p* < 0.01], but not for interactions for the BAI. At Stage 1, the Moderate group had higher scores than the Controls. At Stages 2 and 3, the Severe and Moderate groups had higher scores than the Controls. At Stage 4, the severe group had higher scores than the Controls.

## 3 Discussion

Baseline (Stage 1) differences between those who developed ME/CFS following and those who did not have already been examined (9). The main finding in the current study relates to the Stage 4 findings, that those who were more affected by IM and were classified as Severe ME/CFS at Stage 3 were more likely to have symptoms 7 years later. Overall, the findings validated the initial hypothesis of this study. Seventy-nine percent of those with Severe ME/CFS 7 years later continued to meet the criteria for ME/CFS. However, only 30% to 25% of those with either Moderate or Persisting symptoms had ME/CFS at the 7-year follow-up. These findings suggests that ME/CFS following IM is an enduring illness, particularly for those who are more severely affected. Previous research has demonstrated that ME/CFS substantially disrupts multiple areas of students’ lives, including physical functioning, academic attendance and performance, and participation in extracurricular activities (33). For example, Krilov et al. (34) reported that just 14% of adolescents with ME/CFS attended school on a regular basis, while Dowsett and Colby (35) identified ME/CFS as the leading cause of extended medical leave among school-aged adolescents.

At the seven-year follow-up, differences persisted between participants with severe ME/CFS and those who had recovered from IM on seven of the 13 symptoms or domains assessed. This pattern indicates that individuals who exhibit the most pronounced responses to EBV-associated IM are likely to experience ongoing symptoms years later. Supporting this, although not a study of IM, a 25-year follow-up study of 25 pediatric ME/CFS patients by Brown, Bell, Jason, and Christos (36) found that even those who showed improvement continued to demonstrate significant deficits on 23 of 25 measured outcomes compared with healthy controls. These findings suggest that although some adolescents with ME/CFS may no longer meet formal diagnostic criteria over time, many continue to experience symptoms and fail to return to their pre-illness level of functioning.

An additional finding of interest was that, among the 13 pre-illness variables evaluated, only three significant differences occurred across the four participant groups. Stressful life events have been implicated as a possible contributor to ME/CFS development in adults following viral infections (37). Prior descriptions of pediatric ME/CFS, such as those by Arav-Boger and Spirer (38), often depict patients as previously active and ambitious, from upper-middle-class families, and with relatives affected by ME/CFS. However, these accounts and other studies of youth (39, 40) may be biased, as they typically drew on small clinical samples from tertiary care pediatric or adolescent centers. Adolescents with limited healthcare access are likely underrepresented in these studies (41). Additionally, predictors of ME/CFS risk and persistence identified in adults (42) may not generalize to younger populations, including adolescents and college students.

An intriguing finding at the seven-year follow-up was that autonomic symptoms assessed by the COMPASS-31, which had differentiated groups at earlier stages, no longer did so. This attenuation does not necessarily indicate resolution of dysautonomia. Rather, autonomic symptoms following post-infectious illness may be most prominent during earlier phases, when physiological instability and symptom fluctuation are greatest. Over time, individuals with chronic illness often adopt behavioral adaptations (e.g., activity pacing, avoidance of orthostatic stressors, medication use) that can reduce the frequency or salience of autonomic symptoms as captured by self-report instruments. Thus, changes in COMPASS-31 scores may reflect symptom modulation or adaptation rather than normalization of autonomic function.

A similar pattern was observed for the Physical Health domain of the SF-36. Although group differences were evident at earlier stages post-IM, these differences were no longer significant at 7 years. This finding may reflect response shift processes common in long-term chronic illness, whereby individuals recalibrate their perceptions of physical functioning relative to adapted lifestyles rather than pre-illness baselines. Importantly, attenuation on these broad functional domains occurred alongside persistent elevations in fatigue severity, symptom burden, and diagnostic persistence among participants with severe ME/CFS. Together, these results suggest that while certain symptom domains may stabilize or attenuate over time, the core illness trajectory, particularly among those with severe ME/CFS, remains largely unchanged.

An important methodological consideration concerns how illness severity was operationalized at the six-month post-infectious assessment (Stage 3). Severity of illness often is defined by the intensity of symptoms and/or by the impact of symptoms on a person’s functioning. While we used validated instruments to assess both symptom intensity and functional status, there is currently no consensus in the ME/CFS literature regarding cutpoints or algorithms for defining illness severity using these measures. Defining severity *post hoc* using symptom scores would therefore require arbitrary thresholds and could limit comparability with prior research.

For that reason, we chose instead to define severity based on diagnostic convergence across established ME/CFS case definitions, a conservative and replicable method for distinguishing participants with more extensive and persistent illness profiles. We were encouraged to do so because several previous studies we conducted found that defining severity in this way was correlated with objective evidence of greater pathophysiology. For example, when classifying patients into moderate versus more narrow severe ME/CFS case definitions, significant differences have emerged between those with severe ME/CFS criteria vs. controls with saliva biomarker of fatigue concentrations of 2 peptide fragments in a pediatric sample (43), more dense interconnected cytokine networks at least 6 weeks prior to the onset of infectious mono (44), more gastrointestinal distress and autonomic symptoms, along with several immune markers (45), and metabolite differences with 97% accuracy to differentiate groups (46). Finally, those defined as “Severe” according to our criteria did indeed prove to have a pattern of enduring impairment. Specifically, participants classified as Severe ME/CFS at 6 months post-IM exhibited markedly higher rates of diagnostic persistence, greater symptom burden, and poorer outcomes at the seven-year follow-up compared with those classified as Moderate or Persisting.

This study has several limitations. Although the initial cohort included over 4,500 students, the subset who developed IM and subsequently ME/CFS was relatively small, which may have limited statistical power for some subgroup analyses. Additionally, between the end of Stage 1 in 2018 and the seven-year follow-up, many participants were exposed to SARS-CoV-2 and may have developed Long COVID, a condition with symptoms that can overlap with ME/CFS (47). By 2022, an estimated 95% of individuals aged 16 and older had SARS-CoV-2 antibodies from prior infection or vaccination (48), and by February 2024, 17.1–18.2% of U. S. adults reported experiencing Long COVID (49). These factors may partly account for the relatively high prevalence of ME/CFS-like symptoms observed in the Control group. Although we attempted to examine outcomes among participants with a history of COVID-19 within the current sample, the group sizes were too small to allow meaningful statistical comparisons. We found 51.5% of the sample had experienced COVID, but there was not a significant difference between COVID status and the 4 groups. When we examined the COVID status among those with ME/CFS in the four groups, this comparison was also not significant (of the 3 with ME/CFS in the control group, 2 had COVID; of the 4 with ME/CFS in the Persisting group, 3 had COVID; out of the 7 with ME/CFS in the Moderate ME/CFS group, 5 had COVID; and out of the 11 with ME/CFS in the severe group, 4 had COVID but for 3 their COVID status was unclear).

The mechanisms underlying post-viral illnesses remain incompletely understood (50). Prospective longitudinal research is critical for elucidating the trajectory of illness following EBV infection. Our continuing longitudinal study of ME/CFS after IM aims to identify patterns that distinguish individuals who go on to develop ME/CFS, and may also provide insights relevant to Long COVID.

## Data Availability

The raw data supporting the conclusions of this article will be made available by the authors, without undue reservation.
